# Probing densified silica glass structure by molecular oxygen and E’ center formation under electron irradiation

**DOI:** 10.1038/s41598-023-40270-x

**Published:** 2023-08-22

**Authors:** N. Ollier, I. Reghioua, O. Cavani, M. Mobasher, A. Alessi, S. le Floch, L. Skuja

**Affiliations:** 1 Laboratoire des Solides Irradiés Ecole Polytechnique, CNRS, CEA\DRF\IRAMIS, Institut Polytechnique de Paris, 91128 Palaiseau Cedex, France; 2 Institut Lumière Matière, Univ. Lyon, Université Claude Bernard Lyon 1, CNRS, 69622 Villeurbanne, France; 3 Institute of Solid State Physics, University of Latvia, 8 Kengaraga Str., Riga, 1063 Latvia

**Keywords:** Materials science, Physics

## Abstract

This study aims to learn more about the structure of densified silica with focus on the metamict-like silica phase (density = 2.26 g/cm^3^) by examining the formation of E’ point defects and interstitial molecular oxygen O_2_ by 2.5 MeV electron irradiation. High-dose (11 GGy) irradiation creates a metamict-like phase and a large amount of interstitial O_2_, which is destroyed upon subsequent additional lower-dose electron irradiation. The O_2_ cathodoluminescence (CL) data indicate that the formation of O_2_ from peroxy linkages Si–O–O–Si in silica network is strongly dependent on the intertetrahedral void sizes. The position and shape of the O_2_ emission line support the idea that the configuration of these voids in metamict phase is close to that of non-densified silica. Moreover, data support the strong correlation between the formation of 3-membered rings of Si–O bonds and E’-centers when silica density increases from 2.20 to 2.26 g/cm^3^.

## Introduction

Silica glass, also known as amorphous silica, continues to serve as indispensable material in many technological applications, such as optical communications, electronics, UV and laser optics, sensor technologies, medicine, materials processing.

The densification of silica glass under high pressure (HP)^1^, by shock waves or irradiation (laser, ions, electrons, neutrons) has been extensively studied^2,3^. Compression of silica gives rise to permanent densification with a densification ratio of 25% (for HP > 15 GPa) while irradiation does not lead to values exceeding 3–4%^3,4^. We recently showed the density convergence to a common “metamict-like” silica phase upon irradiating amorphous silica samples having different initial densities with up to 11 GGy dose of 2.5 MeV electrons^5^. The metamict-like densified silica phase has density of 2.26, and its structure shows a large amount of 3-membered rings, as indicated by the intense 606 cm^-1^ (“D_2_”) band in the Raman spectrum^5^. Those characteristics are very similar to those of the metamict phase obtained from quartz crystal, amorphized by ~ 2 × 10^20^ neutrons/cm^2^^6^. This metamict-like phase could be considered as a “Medium Density Amorphous” (MDA, density = 2.26) phase in addition to the Low Density Amorphous (LDA) and High Density Amorphous (HDA) phases identified under High Pressure experiments^7^. The existence of LDA and HDA phases, and their coexistence is widely discussed, e.g., the transition from HDA to LDA during annealing of densified silica phase^7^. Hence, questioning the positioning of metamict-like phase relative to HDA and LDA presents an interesting aspect towards the still open question of polyamorphism in silica glass^8^. Indeed, the structure and properties of the metamict-like phase are not known in detail, mostly because it's creation is expensive and time-consuming (over 10 days in electron accelerator or several months of neutron irradiation in reactor). Moreover, it has been demonstrated by Hobbs, using electron diffraction measurements^9^ that the structure of metamict phase is slightly different, when obtained from different polymorphs—quartz, cristobalite or tridymite, or when obtained by different irradiations—fast neutrons or electrons. This shows that there is no single unique metamict state of SiO_2_ and that the problem is more complex.

Silica glass and almost all crystalline polymorphs of SiO_2_ are built from corner-sharing SiO_4_ tetrahedra of roughly the same size. These phases are distinguished by different connection topologies of SiO_4_ units. Hence, different phase densities are caused by differently sized inter-tetrahedral (interstitial) voids. In the case of silica glass, these voids cause density fluctuations and Rayleigh scattering. The latter is identified as presently the main obstacle to further reduction of losses in optical communication waveguides and provides for direct applied interest in densified forms of amorphous silica^10^. If the interstitial voids are sufficiently large, they can accommodate oxygen molecules O_2_, formed in irradiated silica glass by dimerization of two interstitial oxygen atoms created in Frenkel process. Whether this process is energetically feasible, it is determined by the size of voids: O_2_ easily forms by 13 MGy γ-irradiation in glassy silica (ρ = 2.20 g/cm^3^) and, in contrast, is completely absent in similarly irradiated α-quartz (ρ = 2.65 g/cm^3^)^11^. There is no measurable O_2_ concentration in α-quartz, even after much higher (7 GGy) dose of MeV electrons^12^. In this way, the creation of radiolytic O_2_ is very sensitive to the void size and can yield information on the changes of configuration of interstitial voids in densified silica glass.

The presence and concentration of interstitial O_2_ molecules in SiO_2_ can be determined by their photoluminescence (PL) peak at 1272 nm (0.974 eV). Concentrations as low as 10^14^ O_2_/cm^3^ can be measured^13^. This PL has reasonably high quantum yield (> 0.1), however, the sensitivity is still limited by the extremely small absorption cross sections (~ 10^–23^ cm^2^^14^) for direct photo-exciting of this spin- and parity-forbidden ^1^Δ_g_ → ^3^∑^-^_g_ emission transition. This problem can be circumvented by measuring in situ cathodoluminescence (CL), instead of PL, where O_2_ is excited by energy transfer by excitons or in recombination processes. O_2_ was detected by CL both in amorphous and crystalline SiO_2_^15^. However, relatively low-energy (30 keV) electrons were used, which deposit energy into a thin surface layer, inducing there unintended effects of surface deformation and a rapid amorphization of SiO_2_ crystal. To avoid this, we perform O_2_ CL analysis of silica samples with different initial densities, using high-energy (2.5 MeV) electrons, which penetrate the samples, and dose range 0–200 MGy, which is much below the amorphization threshold.

In addition to formation of O_2_, we studied the formation of Si dangling bonds ≡Si·, (commonly denoted as “E’ centers") in densified silica glasses. The splitting of the hyperfine ^29^Si doublet of the E’ center in electron paramagnetic resonance (EPR) spectra, characterized by isotropic hyperfine coupling constant A_iso_, can be used to probe the local order around this point defect^16^. Devine et al.^17^ studied the point defect formation in densified silica and their recombination under thermal annealing. They concluded that formation of E’-centers is enhanced due to the existence of strained bonds in densified glass. Further, they observed a large difference between the thermal annealing-induced conversion of E’-centers ≡Si· to peroxy radicals ≡Si − O − O·, when measured in non-densified and densified silica. The rate-limiting factor for this conversion is the diffusion of radiation-induced interstitial O_2_ molecules in silica. Hence it could be deduced that diffusion coefficient of O_2_ at 450 °C in densified silica samples is between 10^7^ and 10^9^ times smaller than that in pristine silica.

The aim of the present work is to obtain additional structural information on pressure-densified silica glasses and radiation-compacted metamict-like silica glass by comparative studies of radiation-induced formation of interstitial O_2_ molecules and silicon dangling bonds (E’-centers) in these materials.

## Results

High purity stoichiometric synthetic silica glass samples of commercial type Suprasil F300 were densified at different pressures and temperatures (Table 1). The samples are labeled as PxTy, where x and y are densification pressure (GPa) and temperature (°C), P0T0 stands for non-densified pristine sample. Two additional samples, pristine one and the most-densified one, were irradiated by 11GGy dose of 2.5 MeV electrons, which significantly increased and decreased their respective densities (Table 1, samples P0T0-11GGy and P5T1000-11GGy). For comparison, oxygen-deficient (type KUVI) and oxygen excess (type Suprasil-W) synthetic silica samples were also investigated. Cathodoluminescence of interstitial O_2_ molecules and its dose-dependence were studied in all samples, using 2.5 MeV electron excitation and accumulated doses between 56 and 199 MGy.

**Table 1:** Characteristics and labeling of the synthetic SiO_2_ glasses studied.

Type of SiO_2_ material	Sample label	Densification pressure (GPa)	Densification temperature (° C)	Pre-irrad. dose	CL meas. dose	Initial density (g/cm^3^)	Final density (g/cm^3^)
Suprasil F300	P0T0	0	0	0	62MGy	2.20	2.204
	P5T350	5	350	0	56MGy	2.422	2.41
	P4T450	4	450	0	100MGy	2.294	2.344
	P5T1000	5	1000	0	199MGy	2.612	2.5817
KUVI	O-deficient	0	0	0	186MGy	2.217	2.202
Suprasil W	O-rich	0	0	0	104MGy	2.214	
Suprasil F300	P0T0 11GGy	0	0	11GGy	+ 103MGy	2.257	2.256
	P5T1000 11GGy	5	1000	11GGy	+ 94MGy	2.282	2.288
α-quartz	α-quartz	–	–	1 × 10^19^n/cm^2^		2.65	

Initial and final densities were measured before and after the irradiation dose accumulated during the cathodoluminescence (CL) measurement. Neutron-irradiated synthetic α-quartz crystal was used in a single experiment to compare O_2_ CL band shapes in glass and crystal.

**Figure 1:**
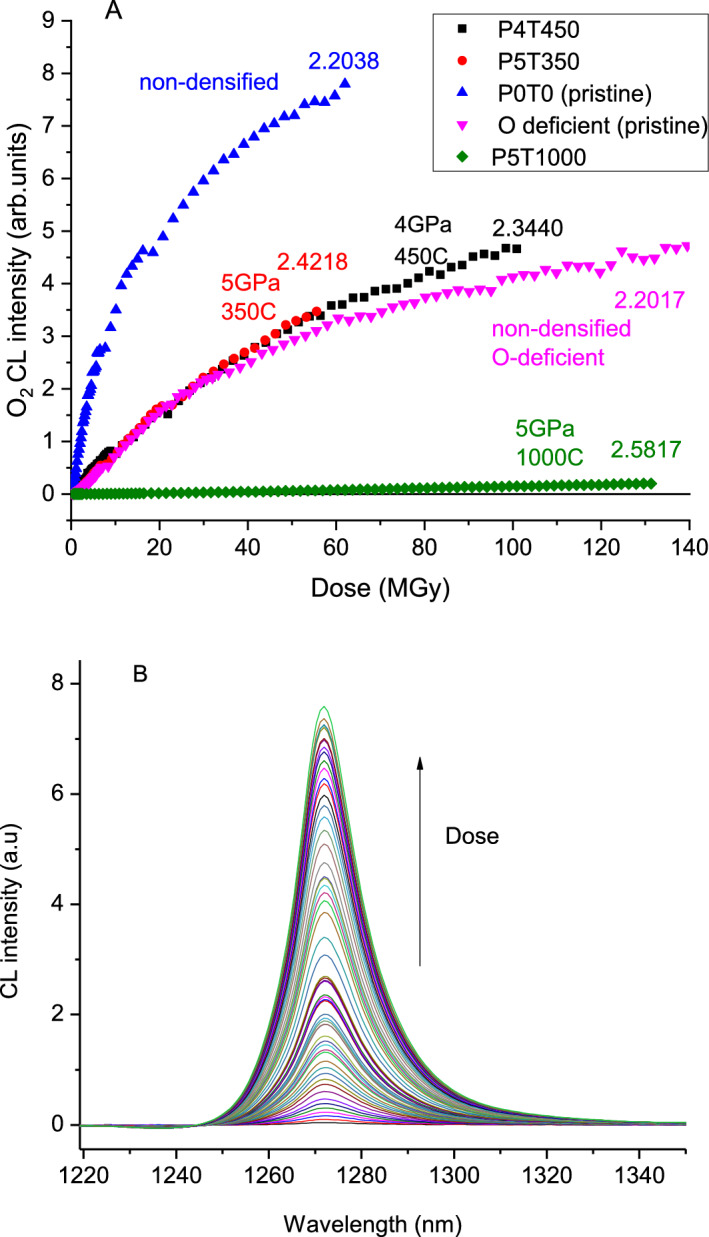
(**A**) Dose-dependence of cathodoluminescence intensity of radiation-induced O_2_ molecules in stoichiometric silica samples, densified at 4 GPa and 5 GPa and T = 350C, 450C and 1000C; and in non-densified stoichiometric and oxygen-deficient samples (see Table 1). The respective densities (in g/cm^3^) measured after the irradiation are indicated. The samples did not contain interstitial O_2_ prior to irradiation. (**B**) Evolution with dose of the ^1^Δ_g_ → ^3^∑^-^_g_ CL emission of O_2_ in non-densified SiO_2_. These spectra provide data-points for the blue (top) curve in panel (**A**).

Figure 1 reports the spectra (panel B) and intensity (panel A) of the interstitial O_2_ CL emission line at 1272 nm as a function of the irradiation dose. Three groups of O_2_ CL intensity growth curves can be outlined, related to the glass density and stoichiometry. In all cases, the intensity is zero at the start of irradiation, and the saturation is not reached at 60 MGy and beyond. The formation of O_2_ is much more efficient in pristine stoichiometric silica than in densified silica glasses or O-deficient one. The curves of densified (ρ = 2.42 and 2.34 g/cm^3^) silica glasses overlap well with the plot of non-densified O-deficient silica (ρ = 2.20 g/cm^3^), whereas the amorphous silica with the highest initial density (ρ = 2.58 g/cm^3^) exhibits a quasi-linear growth with a much lower O_2_ formation rate. The initial slopes of the curves are proportional to the rate of radiolytic formation of interstitial O_2_ molecules. The slope for non-densified stoichiometric silica (sample P0T0) is 10 times larger than that for medium-densified silica (P5T350) and 250 times larger, compared to the highly densified silica (P5T1000).

**Figure 2:**
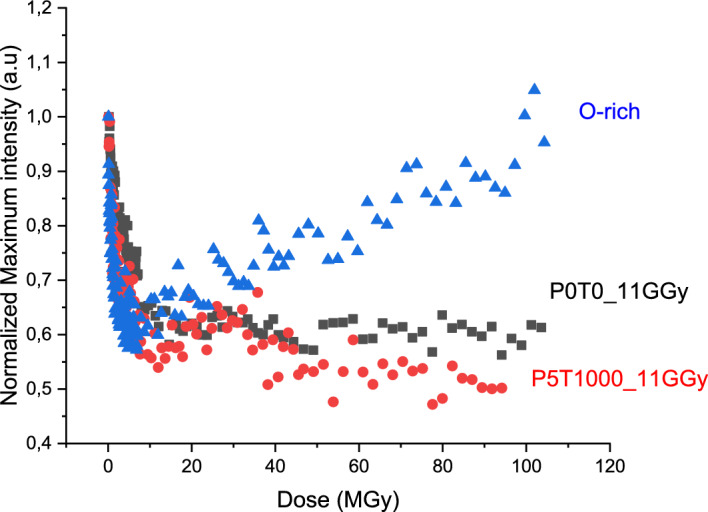
Dose-dependence of O_2_ cathodoluminescence intensity (normalized to 1) in silica samples, containing O_2_ before the start of CL measurement: radiolytic O_2_ (“P0T0_11GGy” and “P5T1000_11GGy”) or synthesis-introduced O_2_ (“O-rich”).

Figure 2 compares the evolution of the O_2_ emission line intensity during irradiation in three samples, all containing sizeable amounts of interstitial O_2_ *before* the start of the irradiation. The pristine “O-rich” silica contained ~ 10^18^ O_2_/cm^3^ introduced by synthesis in oxygen plasma. The two other, initially stoichiometric samples contained interstitial O_2_ introduced by high-dose (11GGy) pre-irradiation. Before the irradiation, one of them was pristine, density 2.21 g/cm^3^_,_ the other was densified to 2.61 g/cm^3^. However, after the 11 GGy irradiation, they both converged to similar density 2.26 g/cm^3^ and thus corresponded to metamict-like silica samples.

It is amazing to observe that CL of those three glasses display a behavior, different from glasses displayed in Fig. 1. A quick decrease of the oxygen peak intensity is observed up to 10 MGy dose, followed by a flat regime for both silica glasses with 2.26 density and a slight increase for the O-rich silica. In metamict-like phases, molecular oxygen has been formed during the irradiation by the dimerization of atomic oxygen created by Frenkel process. There is a general consensus (e.g.,^18,19^) that the ejected O atoms associate with a "regular" bridging O atom in SiO_2_ network and form peroxy linkages (POL): ≡Si − O − O-Si≡. Molecular oxygen forms from 2 POLs, if neighbored by an interstitial void of a sufficient size:

1 $$\equiv {\text{Si}} - {\text{O}} - {\text{O}} - {\text{Si}} \equiv + \equiv {\text{Si}} - {\text{O}} - {\text{O}} - {\text{Si}} \equiv \to {\text{O}}_{{2}} + {2}\left( { \equiv {\text{Si}} - {\text{O}} - {\text{Si}} \equiv } \right).$$

The initial decrease of O_2_ CL intensity in O_2_-rich non-pre-irradiated sample (Fig. 2) points to the radiolysis of interstitial O_2_, which is subsequently equilibrated and superseded by the radiolytic O_2_ formation (Eq. 1), leading to the linear growth of CL at higher doses. When the rates of formation and radiolysis of O_2_ become equal, an equilibrium in O_2_ CL intensity is reached, the equilibrium level depending on the void size.

In the case of both metamict 11 GGy-preirradiated samples, this equilibrium O_2_ level should have been reached during the long pre-irradiation, and is likely to be reproduced by a following much lower dose irradiation. The initial decrease of O_2_ CL intensity evident in Fig. 2 then indicates the amount of additional O_2_ which has formed above that equilibrium level after the cessation of pre-irradiation. It corresponds to the number of POLs, which are spaced close enough to diffuse in SiO_2_ network and recombine at room temperature.

We compared the shape of the emission lines of densified P5T1000 and non-densified SiO_2_ P0T0 irradiated at 11 GGy, measured at the beginning of the CL measurement and at the end (after 100 MGy). The shapes of the four spectra perfectly overlap meaning that there is no evolution of the O_2_ environment after an additional 100 MGy irradiation, and that in both 11 GGy irradiated glasses the molecular oxygen gets a comparable environment.

**Figure 3:**
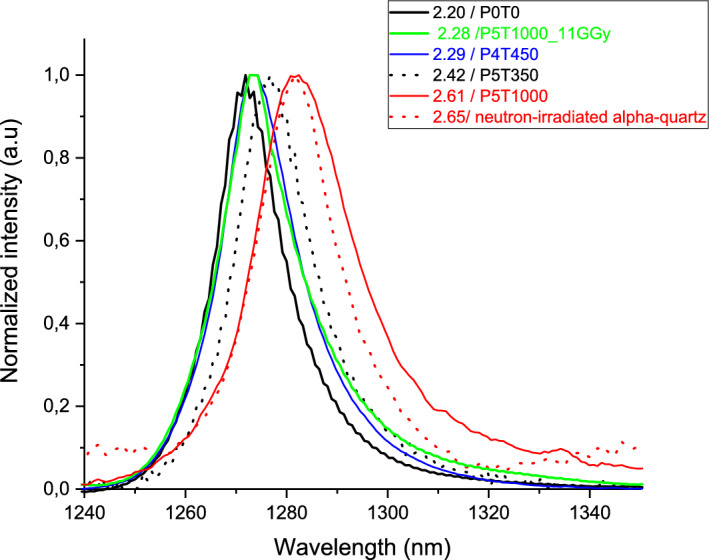
Cathodoluminescence emission spectra of radiolytic O_2_ in electron-irradiated densified and non-densified silica glasses, and neutron-irradiated α-quartz, normalized to 1 for shape comparison. The respective densities (g/cm^3^) are indicated in the legend.

Figure 3 compares shapes of the O_2_ CL emission peaks in a non-densified sample, 3 differently densified samples, and densified/pre-irradiated sample. O_2_ CL band in neutron-irradiated quartz crystal, measured under the same conditions, is displayed for comparison.

O_2_ emission line peaks at 1271.9 nm both for pristine silica and for O-rich sample (an identical spectrum, not shown). The peak red-shifts to 1273.5 nm for slightly-densified samples (ρ = 2.28 and 2.29), while larger red shifts occur for the three highest-density samples: to 1276.7 nm for P5T350 and to 1281.6 nm for P5T1000 and neutron irradiated quartz. The red-shift of the peak position thus follows the density increase.

Compared to pristine silica glass (P0T0), an asymmetric broadening of the CL peak is visible for all samples, even for those with densities of 2.28 and 2.29; it is the largest for P5T1000 sample (FWHM = 23 nm) against 18 nm for quartz and 15 nm for P0T0.

**Figure 4:**
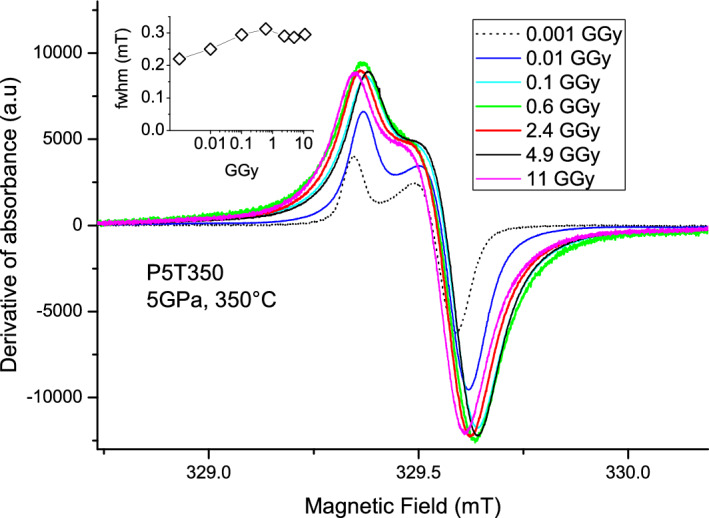
EPR spectra of E’ center in densified sample P5T350 irradiated at doses increasing from 0.001 to 11 GGy. Inset shows the evolution of integrated signal halfwidths (fwhm) with increasing dose.

Figure 4 shows the EPR spectra of E’-centers in P5T350 glass irradiated at step-wise increasing doses. Signal width was evaluated by integrating the derivative signals and determining their full widths at half maximum (fwhm). Figure 4 inset shows that the signal broadens with dose until reaching 0.1 GGy. While this pertains to P5T350 glass, we checked that this result is more general and occurs for all densified glasses. Moreover, the spectra of E’ centers of all 11 GGy-irradiated samples nearly coincide. The broadening of the shape can occur due to the dipolar interaction between the paramagnetic centers getting closer, when their concentration increases, and/or due to the densification of the glass. However, the density of the densified silica glasses is not changed by irradiation up to 1 GGy, and it decreases, if larger than 2.26 g/cm^3^, for doses exceeding 1 GGy, as reported in^20^. Therefore, the broadening should be due to dipolar coupling. Indeed, both the signal width (Fig. 4, inset) and E’-center concentration (Fig. 6) saturate in densified glasses above doses 0.1 GGy.

**Figure 5:**
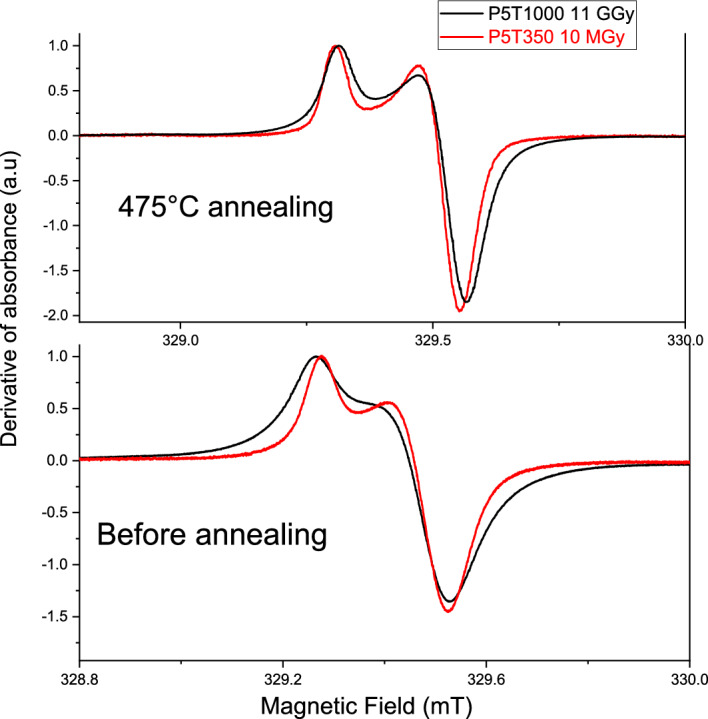
EPR spectra of E’ centers in densified sample P5T350 (ρ = 2.42 g/cm^3^) irradiated by 10 MGy and the metamict sample P5T1000_11GGy (ρ = 2.28 g/cm^3^) before (bottom) and after annealing at 475 °C (top). The spectra are independently normalized to 1.

This conclusion is further supported by thermal annealing data. Figure 5 compares the shapes of E’-center EPR signals and their changes due to thermal annealing at 450 °C in metamict P5T1000_11GGy sample and the densified P5T350 sample irradiated by 10 MGy.

Before annealing, the broadening of the spectrum of 11 GGy-irradiated sample is larger, whereas the sample density is lower compared to P5T350. After annealing at 475 °C, which eliminates ~ 80% of all E’-centers, the signal broadening decreases in both samples and becomes nearly the same. This result confirms that the main origin for the broadening could be the E’-center concentration increase and dipolar effect, rather than the densification, which remains stable upon annealing at 475 °C, as indicated by Raman spectra and density measurements^5^.

**Figure 6:**
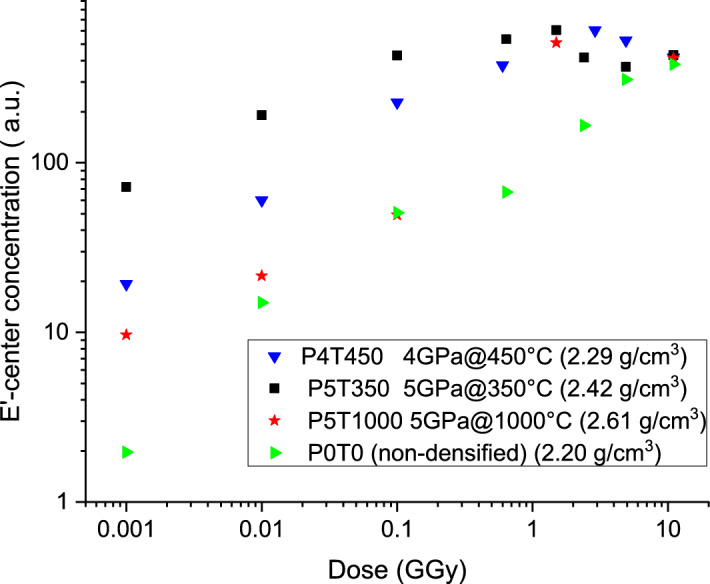
Concentration of E’ center as a function of integrated dose in non-densified and densified glasses.

Figure 6 displays the evolution of the E’ center concentration in the 0–11 GGy dose range for P0T0 (non-densified) and densified P4T450, P5T1000 and P5T350 glasses. Since the shape of the E’-center signal changes with dose, double integration of the signal was used to evaluate the center concentration, instead of the peak-to-peak intensity. It is evident that non-densified glass exhibits a different growth curve compared to densified glass. It reaches saturation concentration of E’-centers around 5–10 GGy, while the saturation dose for densified glasses is around 1 GGy. At low dose, the densified glasses show higher E’-center creation rates, which anti-correlate with densification temperature: defects are created most efficiently in the sample P5T350, densified at the lowest temperature 350 °C.

## Discussion

Figure 6 shows that the formation of E’ center in densified silica is more efficient than in silica in agreement with Devine results^17,21^. He measured a factor 100 in a sample compacted at 5 GPa, 600 °C and irradiated at 1 MGy^17^, while our data shows a maximum ratio of 40 for the 5 GPa, 350 °C at the same dose. The results of both studies indicate that strained bonds lead to a more efficient creation of E’ point defects by bond cleavage, like suggested by Kajihara et al.^13^. The ratio between E’-center concentrations in densified and pristine samples decreases, when dose increases (Fig. 6). At the first glance, this could be related to the changes in density, since at high doses the density of all samples converges to a common, metamict state density value.

However, Fig. 6 illustrates that E’ kinetic growth with dose is not directly dependent on the densification degree, but maybe, on the densification temperature. It is thus worth noticing that a more strained bond, i.e., in a denser silica glass with a lower Si–O-Si angle does not imply a higher efficiency to produce E’ (i.e., P5T1000 vs P4T450). Moreover, it can be underlined that all silica glasses converge towards the same amount of E’ centers, when they reach the same “metamict” phase at 11 GGy. The growth of point defects in dense silica is fast and reaches a maximum of E’ centers at 0.6 GGy whereas in this [0–0.6 GGy] dose range, we showed that the density and the glass network of all densified silica glasses remain constant^5^. We remind that from 1 to 11 GGy, the glass network of dense silica evolves towards a less dense glass with a steady increase of threefold ring amount^5,20^ while the production of E’-centers has saturated. It means that there is no direct correlation between the concentrations of E’-centers and of 3-membered rings, when density is higher than 2.26. This result tends to confirm Devine suggestion^22^ implying that “once the strained bond is cleaved (during the first steps of irradiation in dense silica), the network may relax to a new equilibrium which does not necessarily involve recombination of the Si—O—Si linkage that has been opened.” (and does not necessary lead to the formation of three- and fourfold rings).

**Figure 7:**
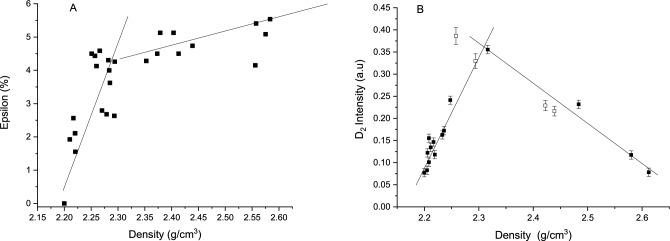
(**A**) Relative change of ^29^Si isotropic hyperfine coupling constant ε = (A_iso_ − A^0^_iso_)/A_iso_ as a function of the density of irradiated samples. The samples are the same as in Fig. 6. (**B**) Raman “D_2_ “– band (threefold-ring) intensity as a function of density (data are taken from ref.^17^).

In addition, the ^29^Si hyperfine structure in the EPR spectrum of E’-centers, and its evolution with the irradiation dose in pristine and differently densified samples P0T0, P4T450, P5T350, P5T1000 (the same as in Fig. 6) was studied. For a clearer analysis, we plotted the parameter ε = (A_iso_ − A^0^_iso_)/ A_iso_ as a function of the silica density (Fig. 7 A), where A_iso_ is the isotropic part of ^29^Si hyperfine coupling tensor. The A^0^_iso_ is a reference value of 41.8 mT that was obtained for a non densified silica irradiated at very low dose in^23^, confirmed as well by our measurements. We clearly see in Fig. 7A, two regimes delimited by the density value 2.30. The first one corresponds to a linear increase of ε in agreement with a strong variation of the local order around the E’ center. Concerning the values, it is worth noticing that the extrapolation of the straight line visible in Fig. 5 in^16^ gives a 5% of epsilon value for the metamict phase identically to ours. Concerning densities higher than 2.26 g/cm^3^, hyperfine constants (A_iso_) are reported in only one paper of Devine^24^. The values from this paper and the evolution of hyperfine versus densification rate are similar to ours when rescaled (The reference A^0^_iso_ from Devine^24^ equals 41 mT instead of 41.8 mT).

It is relevant to mention that a qualitatively similar “two-step” density dependence involving 3-membered rings has been already evidenced in^20^. These previous data on densified silica irradiated at different doses are presented in Fig. 7B. They show the evolution of the D_2_ Raman band intensity as a function of the density with 2 regimes, a linear one up to 2.26. The increase of the 3-membered-ring concentration in the 2.20–2.26 region seems to be clearly associated to local rearrangement after Si–O-Si bond breaking reflected by the increase of the ^29^Si primary hyperfine parameter of the E’ center.

Analyzing the formation of molecular oxygen, we evidenced by our CL measurement that the amount of produced molecular oxygen in densified silica is much lower than in SiO_2_.

Even if the Frenkel process predominates in SiO_2_, with the larger number of strained bonds in densified silica, the bond dissociation mechanism part increase could explain why the E’ number increases so much whereas the O_2_ formation decrease.

One way also to explain the low amount of produced O_2_, particularly in P5T1000 (initial slope is 250 times lower compared to silica) is considering the microstructure of the dense silica. The reduction of the size voids was attested in densified samples by Positron Annihilation Spectroscopy (PAS)^25^. A linear decrease of the void volume with density was evidenced. For a 22% increase of density, it reaches less than 10 Å^3^ against 65 Å^3^ for silica. The density of P5T1000 glass is close to quartz 2.61 compared to pristine quartz 2.65. Indeed, the reduced void size limits the creation of O_2_ that occurs from 2 POL (peroxy linkages (see Eq. 1) that requires sufficiently large voids in order to be formed.

As a matter of fact, the peak position displayed by a neutron irradiated α-quartz studied in^11^ and measured during the same CL experiment is overlapped with the P5T1000. The only difference concerns the larger broadening of the P5T1000 due to a larger variety of O_2_ environment compared to quartz (not amorphized into metamict phase at 10^19^ neutrons/cm^2^).

All 11 GGy irradiated samples display the same characteristics in terms of E’ center (same line shape and amount, and the same hyperfine coupling parameters). It is also important to notice that the undensified sample P0T0 and densified P5T1000 sample after irradiation at 11 GGy show the same O_2_ curve kinetic (Fig. 2) and almost the same emission shape. This confirms that the local order is similar in both samples whatever their different initial structure in addition to the medium range evidenced by Raman spectroscopy in^5^. The fact that O_2_ is destroyed like in O-rich silica means that a large amount of O_2_ is created in the metamict phase inside the voids. This could explain the similarity with the O-rich silica behaviour. Another study by PAS^26^ described a large open structure with an estimated average size of microvoids close to 0.3 nm in silica and metamict phase obtained from quartz amorphization by neutrons. The position and shape of the O_2_ emission line, overlapping with P4T450 sample displaying a 2.29 density supports this (Fig. 3). Indeed, it means that the environment of O_2_ (in links with the voids size) is not so different than in silica and in P4T450 silica glass. Further analyses are needed to determine precisely the metamict phase structure, a ^17^O and ^29^Si NMR ones are undergoing.

## Methods

Synthetic silica glass samples of Suprasil F300 type (< 1 ppm of OH, 2000 ppm Cl) were studied. Some samples were densified before irradiation by using High Pressure, High Temperature (HP-HT) belt press. The experimental details for the HP-HT samples are reported elsewhere^5^. Pressure of 4 GPa was applied at 450 °C and 5 GPa was applied at temperatures 350 and 1000 °C. The obtained samples are labeled as P4T450, P5T350 and P5T1000. O-rich and O-deficient silica samples were also irradiated. The pristine “O-rich” silica contained ~ 10^18^ O_2_/cm^3^ introduced by synthesis in oxygen plasma. The neutron-irradiated synthetic α-quartz crystal had 6 × 10^16^ O_2_/cm^3^^11^.

Density measurements were performed at Institut de Physique du Globe de Paris. Density was measured using the sink-float method based on the Archimedean technique following the law (density (T) = 0.8845–0.9159 × 10^−3^ × T + 0,368.10^−6^ × T^2^, T in °C). The samples were weighed in air and immersed in toluene, which was the immersion liquid used. The sample characteristics are displayed in Table 1.

Near infrared cathodoluminescence spectra were obtained under a 2.5 MeV electrons excitation provided by SIRIUS accelerator (LSI) with a home-made system^27^. The detection was performed by an InGaAs ANDOR camera-based spectrograph operating between 900 nm and 1.6 µm (spectral resolution = 0.14 nm).

The developed Matlab code for extracting the peak intensity of the 1272 nm emission line of O_2_ **(**^1^Δ_g_ → ^3^∑^-^_g_) consists of correcting, automatically, all the obtained spectra from the artefact of the ANDOR camera. For each spectrum, we perform the correction following two steps. First, we subtract the baseline of the intensity at the range around 1400 nm, since the optical fiber transmitting the signal is not transparent at this range (due to the O–H absorption). After that, we normalize the spectra to the intensity of Cherenkov emission in the shortwave region at around 1160 nm.

EPR measurements were carried out at room temperature with a X-Band JES-X310 JEOL spectrometer working at frequency of 9.2 GHz and with magnetic-field modulation frequency of 100 kHz detecting the E’ signal in the conventional, “unsaturated” signal mode (1 µW) and in high-power second harmonic mode (50 mW) to detect the ^29^Si hyperfine doublet.

## Data Availability

The data that support the findings of this study are available from corresponding authors upon request.

## References

[1] Rouxel T, Ji H, Hammouda T, Moréac A (2008). Poisson’s ratio and the densification of glass under high pressure. Phys. Rev. Lett..

[2] Piao F, Oldham WG, Haller EE (2000). Ultraviolet-induced densification of fused silica. J. Appl. Phys..

[3] Ollier N, Girard S, Peuget S (2021). Radiation effects in glass. Encycl. Glass Sci. Technol. Hist. Cult..

[4] Guerette M, Ackerson MR, Thomas J, Yuan F, Bruce Watson E, Walker D, Huang L (2015). Structure and properties of silica glass densified in cold compression and hot compression. Sci. Rep..

[5] Reghioua I, Lancry M, Cavani O, Floch SL, Neuville DR, Ollier N (2019). Unique silica polymorph obtained under electron irradiation. Appl. Phys. Lett..

[6] Bates JB, Hendricks RW, Shaffer LB (1974). Neutron irradiation effects and structure of noncrystalline SiO_2_. J. Chem. Phys..

[7] Guerette M, Ackerson MR, Thomas J, Watson EB, Huang L (2018). Thermally induced amorphous to amorphous transition in hot-compressed silica glass. J. Chem. Phys..

[8] Machon D, Meersman F, Wilding MC, Wilson M, McMillan PF (2014). Pressure-induced amorphization and polyamorphism: Inorganic and Biochemical Systems. Prog. Mater Sci..

[9] Hobbs LW (1995). The role of topology and geometry in the irradiation induced amorphization of network structure. J. Non-Cryst. Solids.

[10] Ono M, Aoyama S, Fujinami M, Ito S (2018). Significant suppression of Rayleigh scattering loss in silica glass formed by the compression of its melted phase. Opt. Express.

[11] Skuja L, Güttler B, Schiel D, Silin AR (1998). Infrared photoluminescence of preexisting or irradiation-induced interstitial oxygen molecules in glassy SiO_2_ and α-quartz. Phys. Rev. B..

[12] Skuja L, Ollier N, Kajihara K, Smits K (2019). Creation of glass-characteristic point defects in crystalline SiO_2_ by 2.5 MeV electrons and by fast neutrons. J. Non-Cryst. Solids.

[13] Kajihara K, Hirano M, Skuja L, Hosono H (2008). Intrinsic defect formation in amorphous SiO_2_ by electronic excitation: Bond dissociation versus Frenkel mechanisms. Phys. Rev. B..

[14] Skuja L, Smits K, Trukhin A, Gahbauer F, Ferber R, Auzinsh M, Busaite L, Razinkovas L, Mackoit-Sinkevičienė M, Alkauskas A (2020). Dynamics of singlet oxygen molecule trapped in silica glass studied by luminescence polarization anisotropy and density functional theory. J. Phys. Chem. C.

[15] Stevens-Kalceff MA (2000). Electron-irradiation-induced radiolytic oxygen generation and microsegregation in silicon dioxide polymorphs. Phys. Rev. Lett..

[16] Buscarino G, Agnello S, Gelardi FM, Boscaino R (2009). Polyamorphic transformation induced by electron irradiation in a-SiO_2_ glass. Phys. Rev. B..

[17] Devine RAB, Capponi JJ, Arndt J (1987). Oxygen-diffusion kinetics in densified, amorphous SiO_2_. Phys. Rev. B..

[18] Ricci D, Pacchioni G, Szymanski MA, Shluger AL, Stoneham AM (2001). Modeling disorder in amorphous silica with embedded clusters: The peroxy bridge defect center. Phys. Rev. B..

[19] Chen Z, Wang J, Song Y, Zuo X (2017). First-principles investigation of oxygen-excess defects in amorphous silica. AIP Adv..

[20] Ollier N, Lancry M, Martinet C, Martinez V, Le Floch S, Neuville D (2019). Relaxation study of pre-densified silica glasses under 2.5 MeV electron irradiation. Sci. Rep..

[21] Devine RAB (1987). Radiation-sensitivity enhancement and annealing variation in densified, amorphous SiO_2_. Phys. Rev. B..

[22] Devine RAB, Arndt J (1989). Correlated defect creation and dose-dependent radiation sensitivity in amorphous SiO_2_. Phys. Rev. B..

[23] Buscarino G, Vaccaro G, Agnello S, Gelardi FM (2009). Variability of the Si–O–Si angle in amorphous-SiO_2_ probed by electron paramagnetic resonance and Raman spectroscopy. J. Non-Cryst. Solids.

[24] Devine RAB, Arndt J (1987). Si–O bond-length modification in pressure-densified amorphous SiO_2_. Phys. Rev. B..

[25] Zanatta M, Baldi G, Brusa RS, Egger W, Fontana A, Gilioli E, Mariazzi S, Monaco G, Ravelli L, Sacchetti F (2014). Structural evolution and medium range order in permanently densified vitreous SiO_2_. Phys. Rev. Lett..

[26] Hasegawa M, Saneyasu M, Tabata M, Tang Z, Nagai Y, Chiba T, Ito Y (2000). Positron and positronium studies of irradiation-induced defects and microvoids in vitreous metamict silica. Nucl. Instrum. Methods Phys. Res. B.

[27] Ollier N, Corbel C, Duchez JB, Cavani O, Benabdesselam M, Mady F (2016). In situ observation of the Yb^2+^ emission in the radiodarkening process of Yb-doped optical preform. Opt. Lett..

